# Genome-wide comparative analysis of photosynthetic enzymatic genes provides novel insights into foxtail millet and other cereals

**DOI:** 10.3389/fgene.2024.1449113

**Published:** 2024-11-05

**Authors:** Arpit Raturi, Shivam Shekhar, Ratnesh Kumar Jha, Divya Chauhan, Saurabh Pandey, Sarita Kumari, Ashutosh Singh

**Affiliations:** ^1^ Department of Agricultural Biotechnology and Molecular Biology, CBS&H, RPCAU-Pusa, Samastipur, Bihar, India; ^2^ Centre for Advanced Studies on Climate Change, RPCAU, Samastipur, Bihar, India; ^3^ Banasthali University, Radha Kishanpura, Rajasthan, India; ^4^ Department of Agriculture, Guru Nanak Dev University, Amritsar, Punjab, India

**Keywords:** C3 photosynthesis, C4 photosynthesis, phylogenetic analysis, gene structure, expression analysis

## Abstract

C4 crops have more efficient photosynthetic pathways that enable their higher photosynthetic capacities as well as nitrogen and water use efficiencies than C3 crops. Previous research has demonstrated that the genomes of C3 species include and express every gene needed for the C4 photosynthesis pathway. However, very little is known about the dynamics and evolutionary history of such genetic evolution in C4 plants. In this study, the genes encoding five key photosynthetic pathway enzymes in the genomes of C3 (rice), C4 (maize, sorghum, and foxtail millet), and CAM (pineapple) crops were identified and compared systematically. The numbers of genes in these photosynthetic enzymes were highest in the C4 crops like sorghum and foxtail millet, while only eight genes were identified in the CAM plant. However, 16 genes were identified in the C3 crop rice. Furthermore, we performed physical, chemical, gene structure and, cis-element analyses to obtain complete insights into these key genes. Tissue-specific expressions showed that most of the photosynthetic genes are expressed in the leaf tissues. Comparisons of the expression characteristics confirmed that the expression patterns of non-photosynthetic gene copies were relatively conserved among the species, while the C4 gene copies in the C4 species acquired new tissue expression patterns during evolution. Additionally, multiple sequence features that could affect C4 gene expressions and subcellular localization were found in the coding and promoter regions. Our research also highlights the variations in how different genes have evolved within the C4 photosynthetic pathway, and we confirmed that specific high expressions in the leaves and right distribution within the cells were crucial for the development of the C4 photosynthetic abilities. The findings of this study are expected to aid in understanding the evolutionary process of the C4 photosynthetic pathway in grasses as well as offer insights for modifying the C4 photosynthetic pathways in wheat, rice, and other significant C3 cereal crops.

## 1 Introduction

Energy flow in the food chain is mainly governed by plants, which use photosynthesis to transform solar energy into biochemical energy, allowing the growth of food for development (Wohlfahrt and Gu, 2015; Shih, 2015). Photosynthesis in higher plants can be divided into three categories as C3, C4, and Crassulacean acid metabolism (CAM) based on how carbon is fixed during the process that produces various initial stable products. The C4 and CAM plants are known to have developed from C3 plants, and most terrestrial plants follow the C3 pathway (Yin et al., 2018). C4 plants are more efficient than C3 plants and very well adapted to subtropical and tropical environments, with lower CO_2_ concentrations and stress environments (Von Caemmerer et al., 2017). C3 plants are involved in carbon fixing through the ribulose bisphosphate carboxylase oxygenase (RUBISCO) enzyme. Meanwhile, the C4 photosynthesis pathway involves numerous enzymes, such as malate dehydrogenase (MD), pyruvate phosphate dikinase (PPDK), nicotinamide adenosine dinucleotide phosphate malic enzyme (NADP-ME), phosphoenolpyruvate carboxylase (PEPCASE), and RUBISCO, which are considered to be the most significant ones (Chen et al., 2014). In C4 plants, PPDK is a crucial enzyme that regulates the pace of photosynthetic activity. Many PPDK genes in C4 and CAM plants, such as *Mesembryanthemum crystallinum* and maize, have been cloned (Fukayama et al., 2001). According to a phylogenetic analysis, the PPDK genes of rice and sorghum are homologous (Wang et al., 2009).

The higher efficiency of C4 photosynthesis is attributed to the enzymes and specific plant morphologies utilizing these enzymes to achieve maximum efficiency. This is accomplished in most C4 plants by spatially separating the Calvin–Benson–Bassham cycle and first CO_2_ fixation between two distinct cell types, which are most frequently mesophyll cells and bundle sheath cells (BSCs) (Hibberd and Covshoff, 2010). The CO_2_ level in the C4 BSCs can be as much as ten times higher than those in the mesophyll cells of C3 plants (Von Caemmerer and Furbank, 2003). Many important crops are known to grow by the C4 pathway, including corn, sorghum, foxtail millet, sugarcane, miscanthus, and switchgrass, and all of these originated in warm and arid climates (Von Caemmerer and Furbank, 2016). Approximately two-thirds of the world’s primary production involves C4 crops, so enhanced photosynthetic capacity has been proposed as the next step toward increasing crop productivity. The C4 photosynthesis pathway is an excellent starting point for increasing the photosynthetic capacities and resource efficiencies of crop plants. Currently, efforts are underway to incorporate C4 pathway traits into C3 crops (Covshoff et al., 2016); the wide variability in the biochemical pathways among C4 species could be attributed to multiple independent evolutions of C4 photosynthesis in distinct plant taxa. The existence of three primary biochemical subtypes among the C4 species has long been recognized, namely phosphoenolpyruvate carboxykinase (PCK), nicotinamide adenine dinucleotide malic enzyme (NAD-ME), and NADP-ME. More recently, it has been proposed that subtypes exist and differ in how well they function under various environmental settings, such as dim light. Given that C4 photosynthesis has evolved at least 25 times in this group of plants, pathway and performance differences are likely to exist, particularly among the grass species to which all C4 cereals belong. Investigating such variations might offer possibilities for enhancing C4 photosynthetic efficiency (Tao et al., 2017). A crop classified as a NADP-ME subtype C4 millet is well-known for its ability to withstand heat waves and drought. In the semi-arid tropical regions of Africa and Asia, it is considered a staple food to more than 500 million people and is a significant source of feed, fiber, and biofuel. Because of these qualities, it is anticipated to become more crucial in addressing the problems of feeding the expanding global population in the face of global warming.

Significant differences have been found in millet in terms of photosynthesis and its associated characteristics, suggesting that the underlying genes are genetically varied (Fernandez et al., 2015). However, minimal information is available on the diversity of photosynthetic genes in millets. In the present study, we investigate the genome-wide photosynthetic genes and compare the genes of the C3, C4, and CAM plants with cis motif analysis, synteny gene ontogeny, and tissue-specific expressions of sorghum and foxtail millet. Furthermore, the study was extended to compare rice, maize, sorghum, and foxtail millet to reveal the evolution of the robust photosynthetic machinery of millet crops.

## 2 Materials and methods

### 2.1 Identification of genes encoding C3, C4, and CAM photosynthetic enzymes in millets

The conserved NADP-ME, PPDK, PEPCASE, RUBISCO, and MD protein sequences were obtained from public databases, namely NCBI (https://www.ncbi.nlm.nih.gov/), Ensembl plants (https://plants.ensembl.org/index.html), and Phytozome (https://phytozome-next.jgi.doe.gov/). The protein and coding sequences (CDSs) for all genes encoding these five enzymes in foxtail millet and sorghum were identified (Kersey et al., 2014). Each of the sequences of sorghum and foxtail millet were compared with the NADP-ME, PPDK, PEPCASE, RUBISCO, and MD protein sequences from rice (*Oryza sativa*) under C3 and maize (*Zea mays*) under C4 using BlastP with a threshold of e-10. The hidden Markov models (HMMs) of the conserved domains of the genes of all five enzymes were obtained from the Pfam database, and BlastP was used to predicted all the genes. Furthermore, the conserved domains were screened using HMMER tools. The NCBI conserve domain database (CDD) was also used to assess the conserved proteins of these candidate genes. The biochemical parameters of NADP-ME, PPDK, PEPCASE, RUBISCO, and MD were calculated from the ExPASy database (https://www.expasy.org/). The subcellular parameters were predicted using Plant-mPLoc (http://www.csbio.sjtu.edu.cn/bioinf/plant-multi/). Finally, the localizations for the five proteins were obtained from the protein FASTA sequences of all the genes of the photosynthetic enzymes and imported into WoLFPSORT. The plants were individually selected as the organism type from the text area under the input method to obtain each gene’s subcellular location. The positions of all the genes and chromosome numbers of the five target enzymes on the chromosome were downloaded from ensemble plants (https://plants.ensembl.org/index.html), and the chromosomal lengths of foxtail millet and sorghum were obtained from NCBI (https://www.ncbi.nlm.nih.gov/). The chromosomal positions and lengths were used to plot graphs with Mapchart v2.2 (Ambadas et al., 2023).

### 2.2 Phylogenetic analysis and identification of C4-orthologous genes in C3 crops

Multiple sequence alignment was performed using ClustalW to evaluate the evolutionary relationships among the genes (https://www.ebi.ac.uk/Tools/msa/clustalo/). The amino acid sequences were first imported to MEGA11 software (https://www.megasoftware.net), and multiple sequence alignment was performed using the default parameters in ClustalW. Then, phylogenetic analyses were conducted using the maximum likelihood (ML) method with 1,000 bootstrap replications, Jones–Taylor–Thornton (JTT) model, and pairwise deletion option (Tamura et al., 2013). The ML trees were constructed using MEGA11 software, and the gene trees of the five proteins were constructed using iTOL v6 software (https://itol.embl.de). MCscanX was used to compare the homology relationships between genes of different species (Wang et al., 2012).

### 2.3 Gene structure and cis-analysis of the promoter region

The gene structures were determined and displayed using the online tool Gene Structure Display Server (GSDS) 2.0 (https://gsds.cbi.pku.edu.cn/). The CDSs and genomic sequences of the identified genes were imported into the GSDS server to identify each of the gene structures for graphical display as well as their intron and exon numbers and regions. Cis-elements of the enzymatic genes of the sorghum and foxtail millet families were determined from a sequence located at the 5′ UTR 2 kb upstream of the sequence considered as the promoter region. The promoter sequences were extracted from the genomic sequences using the Ensembl plants (https://plants.ensembl.org/index.html) and Phytozome (https://phytozome-next.jgi.doe.gov/) databases. The cis-acting regulatory elements in the promoter region were predicted using PlantCARE (https://bioinformatics.psb.ugent.be/webtools/plantcare/html/). The graphs depicting the positions of the regulatory elements were plotted using TBtools software.

### 2.4 Collinearity and synteny analyses

Synteny and collinearity analyses were performed on foxtail millet, sorghum, maize, and rice. The complete genome sequences of foxtail millet, sorghum, maize, and rice were downloaded in the gene finding (gff3) format. The genomic files retrieved from the databases were subjected to the MCScanX tool in TBtools. The dual synteny plotter for MCScanX was used to visualize the results and analyze the syntenic relationships among foxtail–maize, foxtail–rice, sorghum–maize, and sorghum–rice. To obtain the protein sequences, BlastP was used with an e-value of 1.0e^−5^.

### 2.5 Tissue-specific expression patterns

To investigate the expression profiles in different tissues, publicly available RNA-seq datasets of four different tissues (root, shoot, spike, and leaf) of sorghum and foxtail millet were downloaded from the NCBI sequence read archive (SRA) (https://www.ncbi.nlm.nih.gov/sra), gene expression omnibus (GEO) database (https://www.ncbi.nlm.nih.gov/gds), Sorghum Functional Genomics Database (http://structuralbiology.cau.edu.cn/sorghum), *Setaria italica* Functional Genomics Database (http://structuralbiology.cau.edu.cn/SIFGD). To compare the tissue-specific expression characteristics of the homologous genes in different species, expression level (EL) values were used to draw heatmaps utilizing GENEVESTIGATOR software (https://plants.genevestigator.com/local_plants/index.jsp). The fragments per kilobase of transcripts per million mapped reads (FPKM) value was retrieved for each gene along with its normalized reads per kilobase million (RPKM) value.

## 3 Results

### 3.1 Identification of key photosynthetic genes in foxtail millet and sorghum

Genes encoding the C3, C4, and CAM photosynthetic enzymes in foxtail millet and sorghum were identified by searching the protein sequences from the databases noted previously. We also searched for the genes of the five specified enzymes in both crops. A total of 24 genes encoding the key photosynthetic enzymes were confirmed through comparisons of the protein sequences of these enzymes with those of rice and maize using BlastP (Table 1). Furthermore, their filtrations were obtained using the Pfam database and domain structures were confirmed with the CDD. These genes were then assigned identifiers based on their positions in the chromosome, and the enzymes encoded in both sorghum and foxtail millet were designated as shown in Supplementary Table S1. Comprehensive analyses of the genes with their identifiers, gene lengths, gene start and end positions, sequence information, protein lengths, molecular weights, theoretical pI, aliphatic indexes, instability indexes, grand average of hydropathicity (GRAVY) values, and subcellular localizations are given in Supplementary Table S2.

**TABLE 1:** Numbers of genes encoding the five key photosynthetic enzymes in C3 rice, C4 sorghum (*Sorghum bicolor)* and foxtail millet (*Setaria italica*), and CAM (Pineapple) plants.

Enzyme	Number of genes			
	Rice	Sorghum	Foxtail millet	Pineapple
Malate dehydrogenase	2	6	4	2
NADP-ME	5	7	7	0
PEPCASE	4	5	5	2
PPDK	4	2	2	0
RUBISCO	3	4	6	2
Total	18	24	24	6

Different genes show variations for the different characteristics in *Setaria*; for example, the gene length for Seit04_PEP-03 is highest (8.4 kb) whereas that for Seit03_RBCO-04 is least (993 bp). In *Sorghum*, Sobi09_PPDK-01 had the maximum gene length of 20.6 kb, whereas Sobi03_RBCO-04 had the least value (673 bp). Similarly, the largest protein among the different *Setaria* photosynthetic enzymes is Seit05_PEP-02 (1,015 amino acids), while the smallest is Seit03_RBCO-02 (168 amino acids). In *Sorghum*, Sobi01_PEP-01 is the largest protein (1,028 amino acids), whereas Sobi05_RBCO-01 (169 amino acids) is the smallest protein. The molecular weight variations in *Setaria* range from 19 to 113.5 kDa, whereas those in *Sorghum* range from 19 to 115.7 kDa. The isoelectric point was calculated using ExPasy, which shows the ranges of pI to be 4.91–9.01 in *Setaria* and 5.46–8.7 in *Sorghum*, with most proteins exhibiting acidic ranges that suggest their affinities toward acidic environments. Instability index analysis of the proteins in *Setaria* revealed variations ranging from 31.17 to 49.05, with most proteins showing values above 40 that indicate their stabilities; for *Sorghum*, these values varied from 24.72 to 52.00, with most proteins showing stability with values more than 40. The individual variations of specific photosynthetic classes are given in Table 2 for both *Setaria* and *Sorghum*. The domain analyses of these photosynthetic enzymes reveal specific domains for different enzymes (Supplementary Tables S3, S4).

**TABLE 2:** Comparative analysis of different key photosynthetic gene classes in sorghum and foxtail millet.

Particular	Sorghum	Foxtail millet
Malate dehydrogenase		
Gene length range (bp)	2,288–5,298	1,729–4,768
Protein length range	332–434	332–358
Maximum intron and exon numbers	Intron – 13 Exon – 14	Intron – 6 Exon – 7
Minimum intron and exon numbers	Intron – 5 Exon – 6	Intron – 6 Exon – 7
Largest gene	Sobi01_MD-05	Seit09_MD-01
Largest protein	Sobi07_MD-03	Seit07_MD-02
Highest pI	8.23 (Sobi09_MD-02)	8.23 (Seit03_MD-03)
Smallest gene	Sobi06_MD-06	Seit07_MD-02
Smallest protein	Sobi01_MD-05	Seit09_MD-01
NADP – ME		
Gene length range (bp)	1,998–5,994	1,410–7,498
Protein length range	188–652	185–639
Maximum intron and exon numbers	Intron – 19 Exon – 20	Intron – 19 Exon – 20
Minimum intron and exon numbers	Intron – 4 Exon – 5	Intron – 6 Exon – 6
Largest gene	Sobi03_NADP-03	Seit05_NADP-05
Largest protein	Sobi09_NADP-04	Seit05_NADP-04
Highest pI	6.47 (Sobi09_NADP-04)	6.37 (Seit05_NADP-06)
Smallest gene	Sobi05_NADP-01	Seit07_NADP-07
Smallest protein	Sobi05_NADP-01	Seit07_NADP-07
PPDK		
Gene length range (bp)	8,527–20,602	7,088–7,652
Protein length range	904–948	815–893
Maximum intron and exon numbers	Intron – 18 Exon – 19	Intron – 17 Exon – 18
Minimum intron and exon numbers	Intron – 17 Exon – 18	Intron – 16 Exon – 17
Largest gene	Sobi09_PPDK-01	Seit09_PPDK-02
Largest protein	Sobi09_PPDK-01	Seit09_PPDK-02
Highest pI	5.57 (Sobi01_PPDK-02)	5.63 (Seit09_PPDK-02)
Smallest gene	Sobi01_PPDK-02	Seit03_PPDK-01
Smallest protein	Sobi01_PPDK-02	Seit03_PPDK-01
PEPCASE		
Gene length range (bp)	5,587–7,926	4,343–8,432
Protein length range	960–1,028	964–1,015
Maximum intron and exon numbers	Intron – 9 Exon – 10	Intron – 9 Exon – 10
Minimum intron and exon numbers	Intron – 9 Exon – 10	Intron – 6 Exon – 7
Largest gene	Sobi03_PEP-05	Seit04_PEP-03
Largest protein	Sobi01_PEP-01	Seit05_PEP-02
Highest pI	6.12 (Sobi01_PEP-01)	5.95 (Seit04_PEP-03)
Smallest gene	Sobi07_PEP-04	Seit05_PEP-02
Smallest protein	Sobi04_PEP-02	Seit04_PEP-03
RUBISCO		
Gene length range (bp)	673–2,930	993–1,445
Protein length range	169–483	168–427
Maximum intron and exon numbers	Intron – 1 Exon – 2	Intron – 3 Exon – 4
Minimum intron and exon numbers	Intron – 0 Exon – 1	Intron – 1 Exon – 2
Largest gene	Sobi.contig_RBCO-02	Seit.contig_RBCO-06
Largest protein	Sobi.contig_RBCO-02	Seit.contig_RBCO-06
Highest pI	8.77 (Sobi05_RBCO-01)	9.01 (Seit03_RBCO-05)
Smallest gene	Sobi03_RBCO-04	Seit03_RBCO-04
Smallest protein	Sobi05_RBCO-01	Seit03_RBCO-01

**FIGURE 1:**
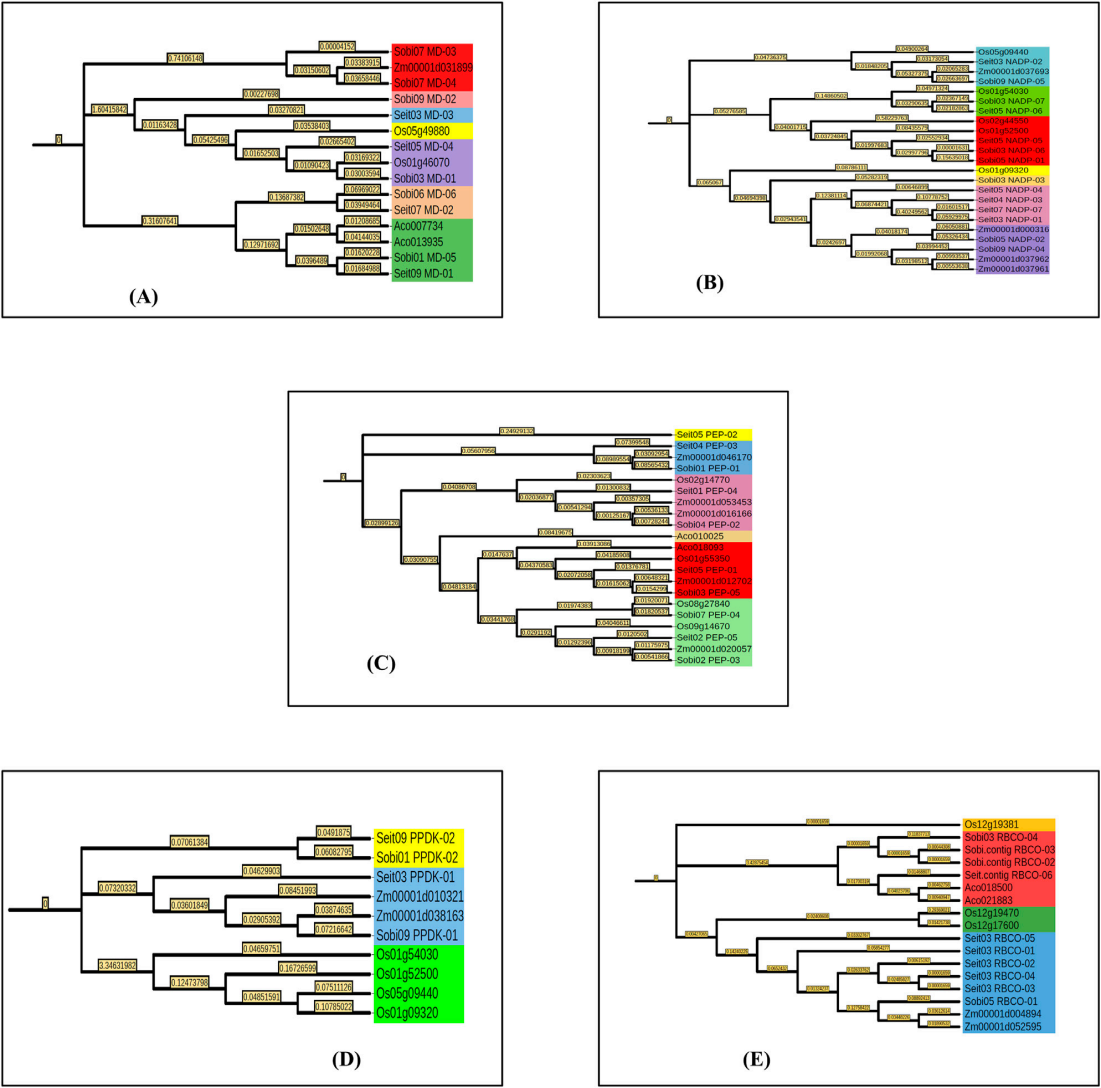
Distribution of key photosynthetic genes on the chromosomes of **(A)** foxtail millet (*Setaria italica*) and **(B)** sorghum (*Sorghum bicolor*). The number of chromosomes is represented above each chromosome.

The chromosomal positions and lengths were used for plotting graphs with Mapchart v2.2. Out of the nine chromosomes, photosynthetic genes were present on seven chromosomes in *Setaria*. Nine genes were maximally present on chromosome no. 3, whereas a minimum of one gene was present on chromosome nos. 1 and 2 in *Setaria*. Similarly, on chromosome nos. 2, 4, 6, and 10, a single gene was present in *Sorghum*, whereas seven genes were maximally present on chromosome no. 3 (Figure 1).

### 3.2 Phylogenetic analyses and evolutionary classifications of the photosynthetic genes of *Setaria* and *Sorghum*

**FIGURE 2:**
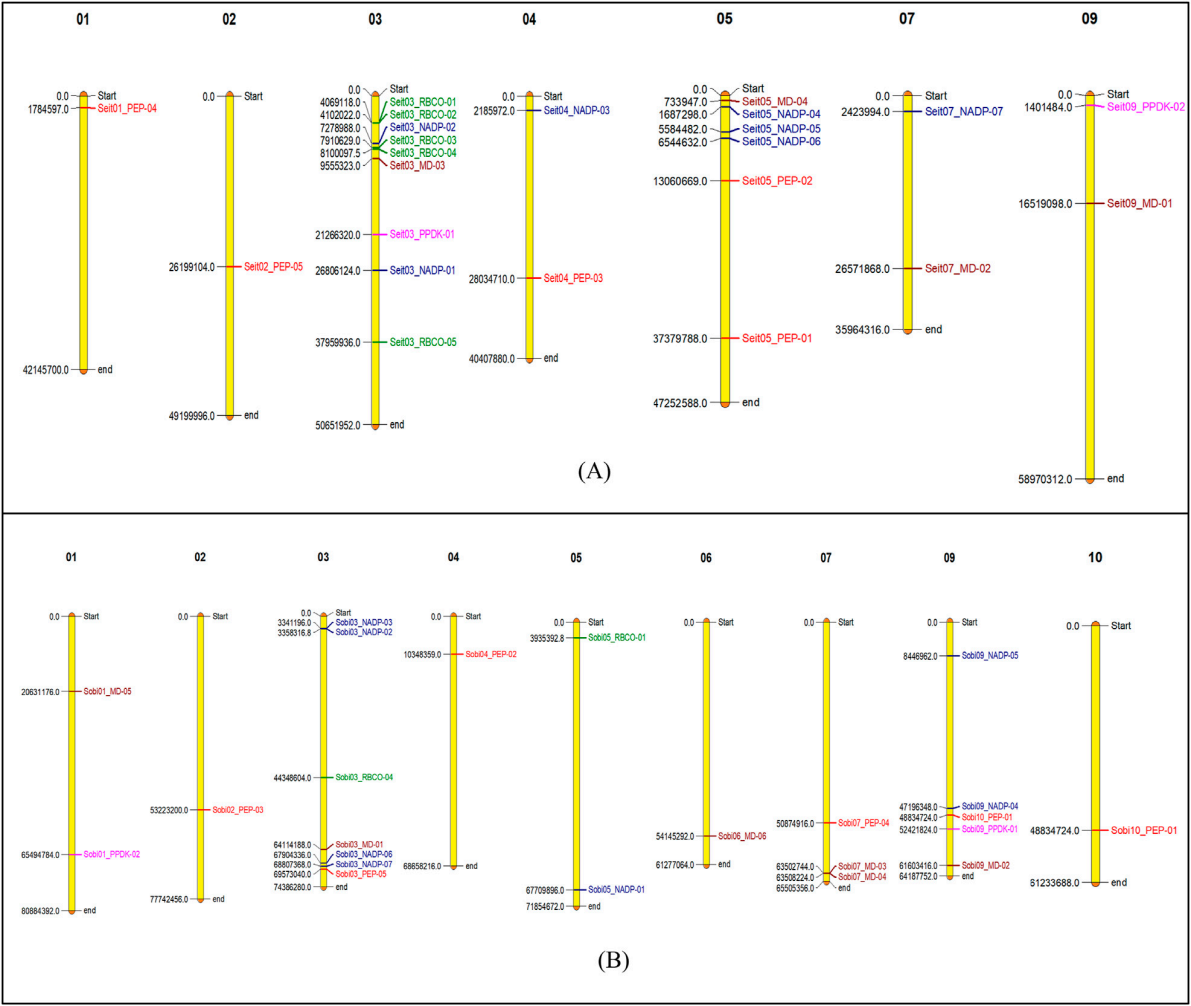
Phylogenetic analyses of foxtail millet, sorghum, rice, maize, and pineapple for key photosynthetic genes: **(A)** malate dehydrogenase, **(B)** NADP-ME, **(C)** PEPCASE, **(D)** PPDK, and **(E)** RUBISCO. Bootstrap tests were performed with 1,000 iterations and are presented at the nodes. The phylogenetic tree is shown on the left side.

Multiple sequence alignments were performed using the genes of foxtail millet and sorghum along with those of rice (C3), maize (C4), and pineapple (*Ananas comosus* var. *comosus*). Phylogenetic analyses were conducted using the ML method and JTT model. The ML trees were constructed using MEGA11 software. MD was found in seven different subgroups, among which subgroup VII had the maximum of four candidates while subgroups II, III, and IV had only single candidates. Similarly, for NADP, seven different subgroups were found, among which subgroups III and VI had the maximum of five candidates while subgroups IV and V had only single members. For PEPCASE, six different subgroups were found, among which subgroup VI had the maximum of six candidates while subgroups I and IV had only single candidates. For PPDK, a total of three subgroups were found, among which subgroup III has four members while subgroup I had two candidates. Interestingly, all the PPDK subgroups of rice were grouped separately from those of the C4 plants. For RUBISCO, a total of four subgroups was noted, among which subgroup IV had the maximum of nine candidates while subgroups I and III had only single members (Figure 2). The biological and molecular functions of all photosynthetic genes were identified, and most of these genes were found to be involved in carbon fixation and biomolecule metabolism. Most of the genes also showed catalytic activities and were required for energy metabolism as well as molecular binding with ATP (Table 3).

**TABLE 3:** Predicted biological and molecular functions of PPDK, PEPCASE, NADP-ME, RUBISCO, and MD in foxtail millet and sorghum along with their gene identities.

S.No.	Gene ID	Biological function	Molecular function
1	Sobi09_PPDK-01	Pyruvate metabolic process and lipid biosynthetic process	Catalytic activity and ATP binding
2	Sobi01_PPDK-02	Pyruvate metabolic process, lipid biosynthetic process, and response to light stimulus	Catalytic activity and ATP binding
3	Sobi01_PEP-01	Tricarboxylic acid cycle and carbon fixation	Catalytic activity, PEPCASE activity, and lyase activity
4	Sobi04_PEP-02	Tricarboxylic acid cycle and carbon fixation	Catalytic activity and PEPCASE activity
5	Sobi02_PEP-03	Tricarboxylic acid cycle and carbon fixation	Catalytic activity and PEPCASE activity
6	Sobi07_PEP-04	Tricarboxylic acid cycle, carbon fixation, and protein tetramerization	Catalytic activity and PEPCASE activity
7	Sobi03_PEP-05	Tricarboxylic acid cycle and carbon fixation	Catalytic activity and PEPCASE activity
8	Sobi03_NADP-02	Malate metabolic process, response to cadmium ions, protein homo-oligomerization, and oxidation–reduction process	Malic enzyme activity, MD (decarboxylating) (NADP+) activity, zinc-ion binding, and oxidoreductase activity
9	Sobi03_NADP-03	Malate metabolic process, response to cadmium ions, protein hom oligomerization, and oxidation–reduction process	Malic enzyme activity, MD (decarboxylating) (NADP+) activity, zinc-ion binding, and oxidoreductase activity
10	Sobi09_NADP-04	Oxidation–reduction process	Oxidoreductase activity, acting on the CH-OH groups of donors, and NAD or NADP as acceptor
11	Sobi09_NADP-05	Malate metabolic process, response to cadmium ions, protein hom oligomerization, and oxidation–reduction process	Malic enzyme activity, MD (decarboxylating) (NADP+) activity, zinc-ion binding, and oxidoreductase activity
12	Sobi03_NADP-06	Malate metabolic process, oxidation–reduction process, and protein hom tetramerization	Malic enzyme activity, MD (decarboxylating) (NADP+) activity, and metal–ion binding
13	Sobi03_NADP-07	Malate metabolic process and oxidation–reduction process	Malic enzyme activity, MD (decarboxylating) (NADP+) activity, and metal–ion binding
14	Sobi05_NADP-01	Response to cold, response to blue light, photorespiration, response to red light, and carbon fixation	Monooxygenase activity, copper-ion binding, and oxidoreductase activity
15	Sobi07_MD-03	Carbohydrate metabolic process, tricarboxylic acid cycle, and malate metabolic process	Catalytic activity, oxidoreductase activity, and MD activity
16	Sobi07_MD-04	Carbohydrate metabolic process and malate metabolic process	Catalytic activity, oxidoreductase activity, and MD activity
17	Sobi01_MD-05	Carbohydrate metabolic process, tricarboxylic acid cycle, malate metabolic process, and response to salt stress	Catalytic activity, oxidoreductase activity, and MD activity
18	Sobi06_MD-06	Carbohydrate metabolic process, tricarboxylic acid cycle, malate metabolic process, and oxidation–reduction process	Catalytic activity, oxidoreductase activity, and MD activity
19	Sobi.contig_RBCO-02	Carbon fixation	Magnesium ion binding and ribulose-bisphosphate carboxylase activity
20	Sobi03_RBCO-04	Carbon fixation, photosynthesis, and photorespiration	Magnesium-ion binding, ribulose-bisphosphate carboxylase activity, lyase activity, and oxidoreductase activity
21	Seit03_PPDK-01	Pyruvate metabolic process and photosynthesis	Nucleotide binding, catalytic activity, ATP binding, kinase activity, and transferase activity
22	Seit09_PPDK-02	Pyruvate metabolic process, photosynthesis, and phosphorylation	Nucleotide binding, catalytic activity, ATP binding, kinase activity, transferase, and metal ion binding
23	Seit05_PEP-01	Tricarboxylic acid cycle, metabolic process, carbon fixation, photosynthesis, and leaf development	Catalytic activity, PEPCASE activity, lyase activity
24	Seit05_PEP-02	Tricarboxylic acid cycle, metabolic process, carbon fixation, photosynthesis, and leaf development	Catalytic activity, PEPCASE activity, and lyase activity
25	Seit04_PEP-03	Tricarboxylic acid cycle, metabolic process, carbon fixation, photosynthesis, and leaf development	Catalytic activity, PEPCASE activity, and lyase activity
26	Seit01_PEP-04	Tricarboxylic acid cycle, metabolic process, carbon fixation, photosynthesis, and leaf development	Catalytic activity, PEPCASE activity, and lyase activity
27	Seit02_PEP-05	Tricarboxylic acid cycle, metabolic process, carbon fixation, photosynthesis, and leaf development	Catalytic activity, PEPCASE activity, and lyase activity
28	Seit03_NADP-01	Pyruvate metabolic process and malate metabolic process	Malic enzyme activity, MD (decarboxylating) (NAD+) activity, MD (decarboxylating) (NADP+) activity, and oxidoreductase activity
29	Seit03_NADP-02	Pyruvate metabolic process and malate metabolic process	Malic enzyme activity, MD (decarboxylating) (NAD+) activity, MD (decarboxylating) (NADP+) activity, and oxidoreductase activity
30	Seit04_NADP-03	Pyruvate metabolic process and malate metabolic process	Malic enzyme activity, MD (decarboxylating) (NAD+) activity, MD (decarboxylating) (NADP+) activity, and oxidoreductase activity
31	Seit05_NADP-04	Pyruvate metabolic process and malate metabolic process	Malic enzyme activity, MD (decarboxylating) (NAD+) activity, MD (decarboxylating) (NADP+) activity, and oxidoreductase activity
32	Seit05_NADP-05	Pyruvate metabolic process and malate metabolic process	Malic enzyme activity, MD (decarboxylating) (NAD+) activity, MD (decarboxylating) (NADP+) activity, oxidoreductase activity, metal ion binding, and NAD binding
33	Seit05_NADP-06	Pyruvate metabolic process and malate metabolic process	Malic enzyme activity, MD (decarboxylating) (NAD+) activity, MD (decarboxylating) (NADP+) activity, oxidoreductase activity, metal ion binding, and NAD binding
34	Seit03_RBCO-02	Photorespiration, carbon fixation, photosynthesis, and reductive pentose-phosphate cycle	Monooxygenase activity and ribulose-bisphosphate carboxylase activity
35	Seit03_RBCO-03	Photorespiration, carbon fixation, photosynthesis, and reductive pentose-phosphate cycle	Monooxygenase activity and ribulose-bisphosphate carboxylase activity
36	Seit03_RBCO-04	Photorespiration, carbon fixation, photosynthesis, and reductive pentose-phosphate cycle	Monooxygenase activity and ribulose-bisphosphate carboxylase activity
37	Seit03_RBCO-05	Photorespiration, carbon fixation, photosynthesis, and reductive pentose-phosphate cycle	Monooxygenase activity and ribulose-bisphosphate carboxylase activity
38	Seit09_MD-01	Carbohydrate metabolic process, tricarboxylic acid cycle, oxaloacetate metabolic process, malate metabolic process, NADH metabolic process, and carboxylic acid metabolic process	Catalytic activity, oxidoreductase activity, MD activity, oxidoreductase activity, acting on the CH-OH groups of donors, NAD or NADP as acceptor, and L-malate dehydrogenase activity
39	Seit07_MD-02	Carbohydrate metabolic process, tricarboxylic acid cycle, oxaloacetate metabolic process, malate metabolic process, NADH metabolic process, and carboxylic acid metabolic process	Catalytic activity, oxidoreductase activity, MD activity, oxidoreductase activity, acting on the CH-OH groups of donors, NAD or NADP as acceptor, and L-malate dehydrogenase activity
40	Seit.contig_RBCO-06	Response to abscisic acid, photorespiration, carbon fixation, photosynthesis, reductive pentose-phosphate cycle, and response to cadmium ions	Magnesium-ion binding, monooxygenase activity, oxidoreductase activity, lyase activity, ribulose-bisphosphate carboxylase activity, and metal ion binding

### 3.3 Photosynthetic enzyme gene structures and cis-element analyses of sorghum and foxtail millet

**FIGURE 3:**
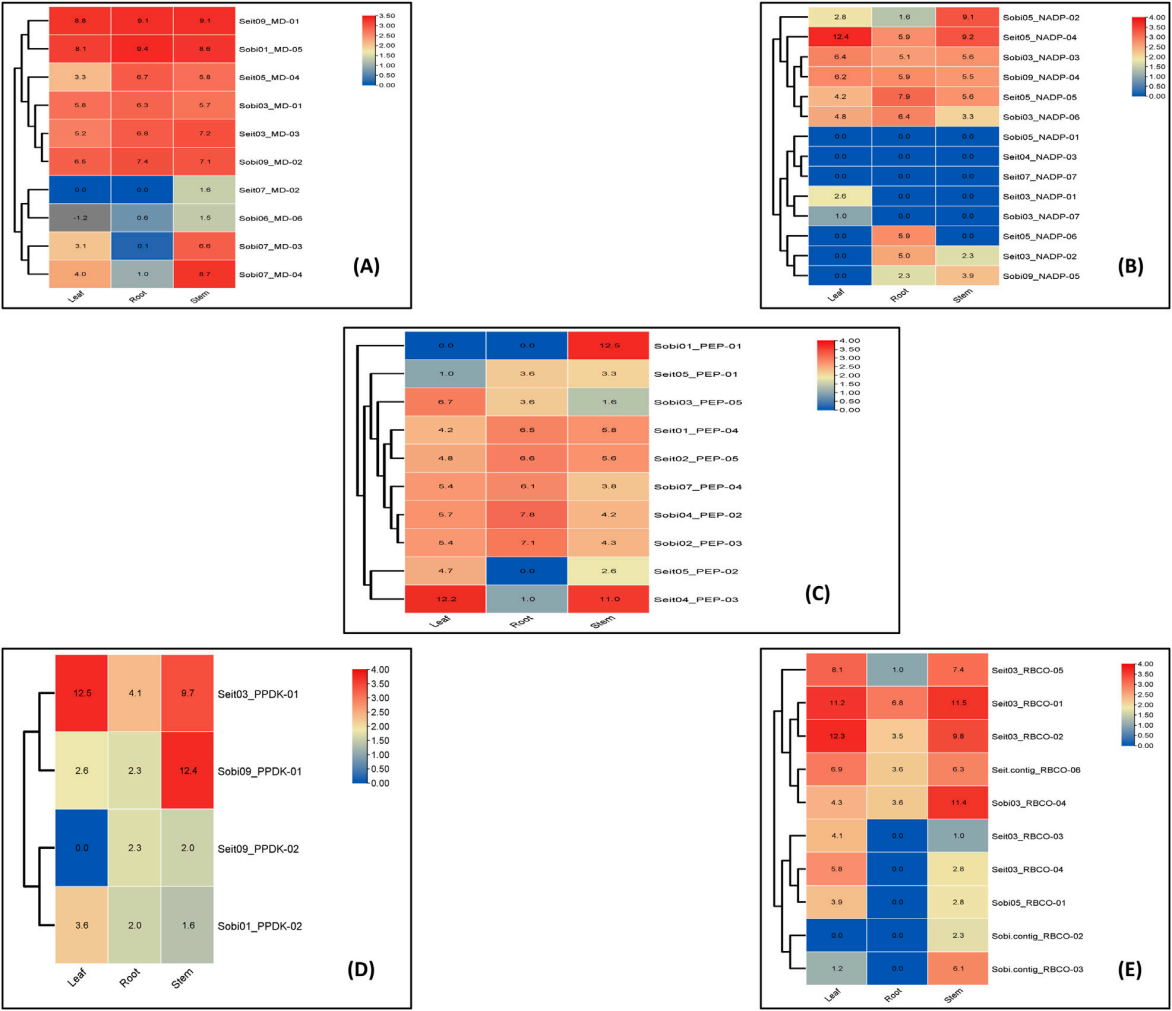
Gene structure analyses: intron/exon structures of the photosynthetic genes of sorghum and foxtail millet. The yellow color represents exon, and the black line represents intron.

The gene structures were determined and displayed using the online tool GSDS 2.0. The gene structures of foxtail millet and sorghum showed variations among the key enzymes (Figure 3). In *Sorghum*, the MD enzyme genes had a maximum of 14 exons and 13 introns (Sobi07_MD-03 and Sobi07_MD-04) along with a minimum of 6 exons and 5 introns (Sobi06_MD-06). However, in all the MD genes of foxtail millet, there were 7 exons and 6 introns present, showing similarity among all MD genes and indicating less evolutionary pressure at the gene structure level in this C4 crop. Similarly, the NADP-ME enzyme genes in sorghum had a maximum of 20 exons and 19 introns (Sobi03_NADP-02 and Sobi09_NADP-04) along with a minimum of 5 exons and 4 introns (Sobi05_NADP-01). The maximum numbers of exons and introns of foxtail millet (Seit05_NADP-04) were similar to those of sorghum, whereas a minimum of 7 exons and 6 introns (Seit03_NADP-01) were present in the gene structures for NADP-ME. The PEPCASE enzyme genes in sorghum all had similar structures with 10 exons and 9 introns, whereas those in foxtail millet had a maximum of 10 exons and 9 introns (Seit01_PEP-04, Seit02_PEP-05, and Seit05_PEP-01) along with a minimum of 7 exons and 6 introns (Seit05_PEP-02). The PPDK enzyme genes in sorghum had a maximum of 19 exons and 18 introns (Sobi09_PPDK 01) along with a minimum of 18 exons and 17 introns (Sobi09_PPDK 01); in foxtail millet, the corresponding maximum numbers were 18 exons and 17 introns (Seit03_PPDK-01) along with a minimum of 17 exons and 16 introns (Seit03_PPDK-02). Out of the four identified genes of the RUBISCO enzyme in sorghum, two have 0 introns and 1 exon (Sobi.contig_RBCO-03 and Sobi.contig_RBCO-02) while the other two have 2 exons and 1 intron each (Sobi03_RBCO_04 and Sobi05_RBCO_01).

**FIGURE 4:**
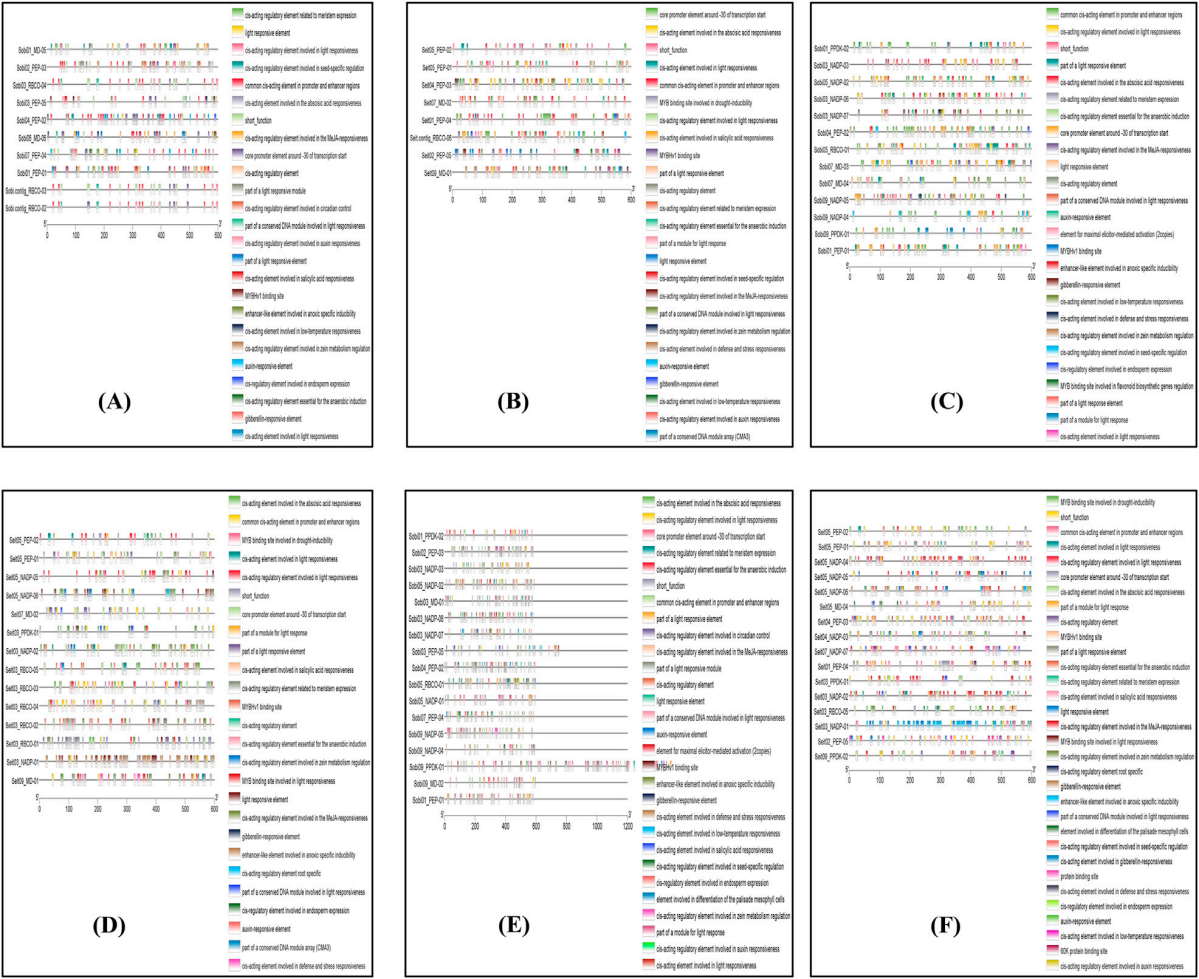
Representation of Cis-element analysis of identified photosynthetic genes in sorghum and foxtail millet.

For the cis-element analysis, the 2 kbp upstream sequence was considered as the promoter region. A graph depicting the positions of the regulatory elements was plotted using TBtools (Figure 4; Supplementary Table S5). These promoter regions of the photosynthetic enzymes are rich in elements, including the light responsiveness elements, transcription factor binding sites, and plant growth regulator responsive elements.

### 3.4 Comparative analyses of the photosynthetic genes of foxtail millet and sorghum with rice (C3) and maize (C4)

**FIGURE 5:**
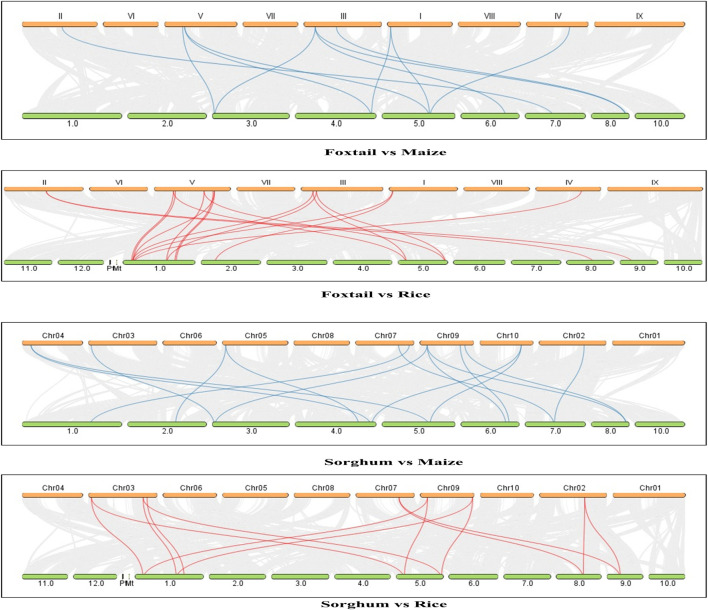
Gene duplication and synteny analyses of the key photosynthetic genes between *Oryza sativa*, maize, sorghum, and foxtail millet.

BlastP and MCScanX tools were used to identify the synteny and collinearity of the essential photosynthetic genes between rice, maize, sorghum, and foxtail millet. Here, foxtail millet and maize showed 47.51% collinearity for the key photosynthetic genes, while 11 pairs of synteny were found between their genomes. Similarly, foxtail millet showed 47.52% collinearity with the rice genome. The proportion of collinear genes between sorghum and rice was 49.50%, with 19 synteny pairs. Meanwhile, sorghum and maize showed 56.05% collinear genes, with 16 pairs of chromosomes showing synteny for these key photosynthetic genes (Figure 5).

### 3.5 Tissue-specific expression patterns of photosynthetic genes in sorghum and foxtail millet

**FIGURE 6:**
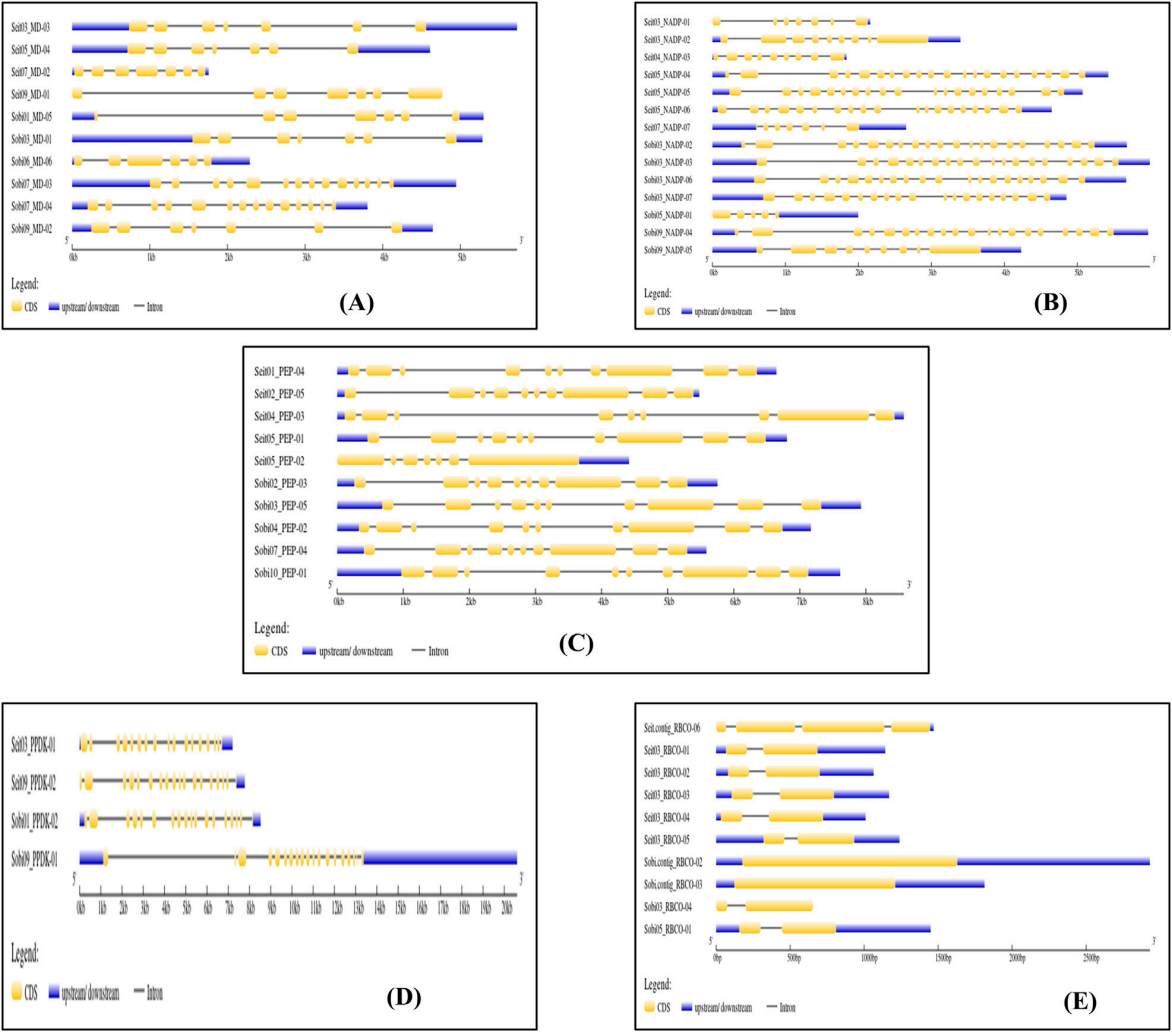
Expression analysis of photosynthetic genes of sorghum and foxtail millet in leaf stem and root tissues **(A)** Malate dehydrogenase **(B)** NADP **(C)** PEPcase **(D)** PPDK **(E)** RuBisCo.

To compare the tissue-specific expression characteristics of homologous genes in different species, EL values were used to obtain heatmaps. For MD, the gene SETIT_036550mg was highly expressed in leaf, root, and stem tissues. Similarly, in *Sorghum*, the gene Sobic.001G219300 was highly expressed in leaf, root, and stem tissues, whereas Sobic.007G166300 was highly expressed only in the crop stem. For NADP, the gene SETIT_000645mg was highly expressed only in foxtail leaf tissues, while the other genes of sorghum and foxtail millet showed moderate or low or downregulated ELs in leaf, stem, and root tissues. The heatmap of PEPCASE showed that the gene Sobic.010G160700 was highly expressed in the stem tissue of sorghum, whereas SETIT_005789mg was highly expressed in the leaf and stem tissues of foxtail millet. For PPDK, the gene Sobic.009G132900 was highly expressed in stem tissue, while SETIT_021174mg was highly expressed in leaf and stem tissues (Figure 6).

## 4 Discussion

C4 photosynthesis is more efficient than C3 that evolved 30 million years ago (Aubry et al., 2011), and most C4 plant species are monocots (Gowik and Westhoff, 2011). Studies have previously shown that the enzymes PEPCASE, NADP-ME, and PPDK were necessary for C4 photosynthesis despite the unique cellular structures of C4 plants (Jiang et al., 2016). In the present study, the total number of photosynthetic genes were found to be higher for C4 crops (sorghum and foxtail millet) and lower in the case of CAM plants (pineapple). These results suggest that C4 plants have more efficient photosynthesis because of their greater diversity of photosynthetic genes. In this study, the number of photosynthetic genes were identified to be less than that reported by Chen et al. (2023) because we considered that the blast similarity percentage should exceed 50% while the e-value used was e-10 instead of e-5 to increase the chances of finding more specific hits. Most of the photosynthesis genes of sorghum and foxtail millet are located in the cytoplasm and chloroplast. The phylogenetic analysis between rice (C3); maize, sorghum, and foxtail millet (C4); and pineapple (CAM) revealed that there is an evolutionary relationship between C3, C4, and CAM plants and that different genes evolved over different periods (Mallmann et al., 2014). The gene lineages PEPCo (4), ME (4), and MDH (3) having origins independent from those of the C4 grass crops were found to have been recruited to the C4 photosynthetic pathway, confirming their identities during C4 evolution and rendering their gene copies in C3 crops as significant candidates for gene manipulation; these findings are consistent with earlier research reports (Moreno-Villena et al., 2018).

Moreover, positive gene duplication events related to the expansion of these gene families were confirmed, with more duplicated genes in the branches with C4 photosynthetic gene copies. Previous studies have suggested that duplication events are the first step of C4 evolution (Sage et al., 2012). It is interesting to note that the NADP-ME and PPDK gene counts increased later in the evolution process. Numerous investigations have indicated that several duplication events transpired throughout the evolution of plants. These include the γ event that distinguished monocots from dicots (Bowers et al., 2003); ρ event that occurred prior to the divergence of wheat, maize, and rice but subsequent to the divergence of grasses and pineapple (Ming et al., 2015); and τ and σ events within the Poaceae family (McKain et al., 2016).

The gene exon–intron structures were studied using gene structure analysis. The number of introns varied between sorghum and foxtail millet. The introns of genes are important structural components that are crucial for regulating gene expressions through enhancer-mediated and alternative splicing processes mediated by introns. These processes increase the efficacy of natural selection. It has been reported that copy-paste is the usual method of creating intron-deficient genes that are most likely the product of retro-transposition (Deshmukh et al., 2016). To obtain insights into the regulation of photosynthetic genes, we examined the promoter regions of significant stress-responsive transcription factors for cis-elements that are known to influence stress-responsive genes. These promoter regions of photosynthetic enzymes are rich in cis-elements, including light responsiveness elements, transcription factor binding sites, and plant growth regulator responsive elements.

Along the development of the C4 photosynthetic pathway, the regulatory areas of the original C3 genes changed or had alterations in the CDSs, leading to shifts in the locations of essential enzymes, noticeable changes in their activities, and specific expression patterns within the cells (Ludwig, 2013). The tissue-specific expressions revealed that most of the photosynthetic enzymes were highly expressed in the leaves while a few were expressed in the stem, suggesting the ELs of key photosynthetic genes in these regions. Mining of natural allelic variations for enhanced photosynthesis requires understanding of the genetic variations of the major enzymes of the C4 pathway. Additional research examining these allelic variations for correlation with agronomic characteristics will offer fresh opportunities for the photosynthetic efficiency enhancement of sorghum and foxtail millet. Multiple elements related to hormone-response-related and meristem-specific expressions were found in the upstream region of the C4 RbcS initiation codon, which may directly regulate the specific expressions of C4 RbcS in the BSCs. In a previous study, it was shown that promoter deletion in the maize RbcS promoter region resulted in deletion of the −211 to +434 regions and accumulation of RbcS in the BSCs, which are consistent with our results (Purcell et al., 1995). However, regional differences exist in the three representative C4 crops considered in this study, confirming that similarly high expressions of C4 RbcS in the BSCs are regulated.

## 5 Conclusion

In this study, the genes encoding five key photosynthetic enzymes of sorghum and foxtail millet were systematically identified, analyzed, and compared with other cereals and pineapple. The total number of key photosynthetic enzymes were equal in sorghum and foxtail millet; this number was similar to that of the C3 crop rice but lowest in the CAM plant pineapple. Phylogenetic analysis showed that C4 crops have a closer evolutionary relationship with C3 crops, and promoter analysis showed that photosynthetic genes influence stress-responsive genes. The promoter regions of the photosynthetic enzymes include light responsiveness elements, transcription factor binding sites, and plant growth regulator responsive elements. The findings of this study also suggest that most photosynthetic genes are expressed in the leaves, while only a few genes are expressed in the stem. Hence, most of the photosynthetic genes are very active in leaf tissues. The findings of this study are expected to contribute toward the advancement of knowledge regarding the C4 photosynthetic conversion of significant C3 species, including major cereal crops such as wheat and rice.

## Data Availability

The original contributions presented in the study are included in the article/Supplementary Material; further inquiries can be directed to the corresponding author.

## References

[B1] Ambadas D. A. Singh A. Jha R. K. Chauhan D. Sharma V. K. (2023). Genome-wide dissection of AT-hook motif nuclear-localized gene family and their expression profiling for drought and salt stress in rice (Oryza sativa). Front. Plant Sci. 14, 1283555. 10.3389/fpls.2023.1283555 38148863 PMC10749976

[B2] Aubry S. Brown N. J. Hibberd J. M. (2011). The role of proteins in C3 plants prior to their recruitment into the C4 pathway. J. Exp. Bot. 62 (9), 3049–3059. 10.1093/jxb/err012 21321052

[B3] Bowers J. E. Chapman B. A. Rong J. Paterson A. H. (2003). Unravelling angiosperm genome evolution by phylogenetic analysis of chromosomal duplication events. Nature 422 (6930), 433–438. 10.1038/nature01521 12660784

[B4] Chen Y. B. Lu T. C. Wang H. X. Shen J. Bu T. T. Chao Q. (2014). Posttranslational modification of maize chloroplast pyruvate orthophosphate dikinase reveals the precise regulatory mechanism of its enzymatic activity. Plant physiol. 165 (2), 534–549. 10.1104/pp.113.231993 24710069 PMC4044839

[B30] Chen L. Yang Y. Zhao Z. Lu Q. Cui C. Parry M. A. (2023). Genome-wide identification and comparative analyses of key genes involved in C4 photosynthesis in five main gramineous crops. Front. Plant Sci. 14, 1134170. 36993845 10.3389/fpls.2023.1134170 PMC10040670

[B5] Covshoff S. Szecowka M. Hughes T. E. Smith-Unna R. Kelly S. Bailey K. J. (2016). C4 photosynthesis in the rice paddy: insights from the noxious weed Echinochloa glabrescens. Plant Physiol. 170 (1), 57–73. 10.1104/pp.15.00889 26527656 PMC4704570

[B6] Deshmukh R. K. Sonah H. Singh N. K. (2016). Intron gain, a dominant evolutionary process supporting high levels of gene expression in rice. J. Plant Biochem. Biotechnol. 25, 142–146. 10.1007/s13562-015-0319-5

[B7] Fernandez M. G. S. Strand K. Hamblin M. T. Westgate M. Heaton E. Kresovich S. (2015). Genetic analysis and phenotypic characterization of leaf photosynthetic capacity in a sorghum (Sorghum spp.) diversity panel. Genet. Resour. Crop Ev. 62, 939–950. 10.1007/s10722-014-0202-6

[B8] Fukayama H. Tsuchida H. Agarie S. Nomura M. Onodera H. Ono K. (2001). Significant accumulation of C4-specific pyruvate, orthophosphate dikinase in a C3 plant, rice. Plant physiol. 127 (3), 1136–1146. 10.1104/pp.127.3.1136 11706193 PMC129282

[B9] Gowik U. Westhoff P. (2011) “Chapter 13 C 4-phosphoenolpyruvate carboxylase,” in C4 photosynthesis and related CO2 concentrating mechanisms, 257–275.

[B10] Hibberd J. M. Covshoff S. (2010). The regulation of gene expression required for C4 photosynthesis. Annu. Rev. plant Biol. 61, 181–207. 10.1146/annurev-arplant-042809-112238 20192753

[B11] Jiang L. Chen Y. B. Zheng J. Chen Z. Liu Y. Tao Y. (2016). Structural basis of reversible phosphorylation by maize pyruvate orthophosphate dikinase regulatory protein. Plant physiol. 170 (2), 732–741. 10.1104/pp.15.01709 26620526 PMC4734583

[B29] Kersey P. J. Allen J. E. Christensen M. Davis P. Falin L. J. Grabmueller C. (2014). Ensembl Genomes 2013: scaling up access to genome-wide data. Nucleic Acids Res. 42 (D1), D546–D552. 24163254 10.1093/nar/gkt979 PMC3965094

[B12] Ludwig M. (2013). Evolution of the C 4 photosynthetic pathway: events at the cellular and molecular levels. Photosynth. Res. 117, 147–161. 10.1007/s11120-013-9853-y 23708978

[B13] Mallmann J. Heckmann D. Bräutigam A. Lercher M. J. Weber A. P. Westhoff P. (2014). The role of photorespiration during the evolution of C4 photosynthesis in the genus Flaveria. Elife 3, e02478. 10.7554/eLife.02478 24935935 PMC4103682

[B14] McKain M. R. Tang H. McNeal J. R. Ayyampalayam S. Davis J. I. Depamphilis C. W. (2016). A phylogenomic assessment of ancient polyploidy and genome evolution across the Poales. Genome Biol. Evol. 8 (4), 1150–1164. 10.1093/gbe/evw060 26988252 PMC4860692

[B15] Ming R. VanBuren R. Wai C. M. Tang H. Schatz M. C. Bowers J. E. (2015). The pineapple genome and the evolution of CAM photosynthesis. Nat. Genet. 47 (12), 1435–1442. 10.1038/ng.3435 26523774 PMC4867222

[B16] Moreno-Villena J. J. Dunning L. T. Osborne C. P. Christin P. A. (2018). Highly expressed genes are preferentially co-opted for C4 photosynthesis. Mol. Biol. Evol. 35 (1), 94–106. 10.1093/molbev/msx269 29040657 PMC5850498

[B17] Purcell M. Mabrouk Y. M. Bogorad L. (1995). Red/far-red and blue light-responsive regions of maize rbcS-m3 are active in bundle sheath and mesophyll cells, respectively. Proc. Natl. Acad. Sci. U.S.A. 92, 11504–11508. 10.1073/pnas.92.25.11504 8524792 PMC40430

[B18] Sage R. F. Sage T. L. Kocacinar F. (2012). Photorespiration and the evolution of C4 photosynthesis. Annu. Rev. plant Biol. 63, 19–47. 10.1146/annurev-arplant-042811-105511 22404472

[B19] Shih P. M. (2015). Photosynthesis and early earth. Curr. Biol. 25 (19), R855–R859. 10.1016/j.cub.2015.04.046 26439346

[B20] Tamura K. Stecher G. Peterson D. Filipski A. Kumar S. (2013). MEGA6: molecular evolutionary genetics analysis version 6.0. Mol. Biol. Evol. 30 (12), 2725–2729. 10.1093/molbev/mst197 24132122 PMC3840312

[B21] Tao Y. Mace E. S. Tai S. Cruickshank A. Campbell B. C. Zhao X. (2017). Whole-genome analysis of candidate genes associated with seed size and weight in sorghum bicolor reveals signatures of artificial selection and insights into parallel domestication in cereal crops. Front. plant Sci. 8, 1237. 10.3389/fpls.2017.01237 28769949 PMC5513986

[B22] Von Caemmerer S. Furbank R. T. (2003). The C 4 pathway: an efficient CO 2 pump. Photosynth. Res. 77, 191–207. 10.1023/A:1025830019591 16228376

[B23] Von Caemmerer S. Furbank R. T. (2016). Strategies for improving C4 photosynthesis. Curr. Opin. plant Biol. 31, 125–134. 10.1016/j.pbi.2016.04.003 27127850

[B24] Von Caemmerer S. Ghannoum O. Furbank R. T. (2017). C4 photosynthesis: 50 years of discovery and innovation. J. Exp. Bot. 68 (2), 97–102. 10.1093/jxb/erw491 28110274 PMC5444450

[B25] Wang X. Gowik U. Tang H. Bowers J. E. Westhoff P. Paterson A. H. (2009). Comparative genomic analysis of C4 photosynthetic pathway evolution in grasses. Genome Biol. 10, R68–R18. 10.1186/gb-2009-10-6-r68 19549309 PMC2718502

[B26] Wang Y. Tang H. DeBarry J. D. Tan X. Li J. Wang X. (2012). MCScanX: a toolkit for detection and evolutionary analysis of gene synteny and collinearity. Nucleic acids Res. 40 (7), e49. 10.1093/nar/gkr1293 22217600 PMC3326336

[B27] Wohlfahrt G. Gu L. (2015). The many meanings of gross photosynthesis and their implication for photosynthesis research from leaf to globe. Plant, cell and Environ. 38 (12), 2500–2507. 10.1111/pce.12569 PMC4681079 25988305

[B28] Yin H. Guo H. B. Weston D. J. Borland A. M. Ranjan P. Abraham P. E. (2018). Diel rewiring and positive selection of ancient plant proteins enabled evolution of CAM photosynthesis in Agave. BMC genomics 19, 1–16. 10.1186/s12864-018-4964-7 30081833 PMC6090859

